# CDC dengue typing kit fails to detect dengue virus 2 sylvatic genotype

**DOI:** 10.1128/jcm.01741-24

**Published:** 2025-07-17

**Authors:** Diamilatou Balde, Mignane Ndiaye, Agathe Shella Efire, Ousmane Faye, Amadou Alpha Sall, Manfred Weidmann, Oumar Faye, Idrissa Dieng

**Affiliations:** 1 Virology unit, Institut Pasteur de Dakar 89248, Dakar, Senegal; 2 Institute of Microbiology and Virology, Brandenburg Medical School 477107 , Senftenberg, Brandenburg, Germany; 3 Animal Biology Department, Universite Cheikh Anta Diop de Dakar 89230 , Dakar, Senegal; Mayo Clinic Minnesota, Rochester, Minnesota, USA

**Keywords:** CDC dengue typing kit, DENV-2/GVI, RT-qPCR, target failure

## LETTER

Dengue is considered as one of the most prevalent arboviral threats worldwide (1). The virus exists in four (DENV-1, DENV-2, DENV-3, and DENV-4) antigenically and phylogenetically distinct forms, namely viral serotypes (2). In Africa, the virus was thought to be rare since it was first reported from the continent in the 19th century (3). The virus epidemiology is marked by circulation in two transmission cycles: the epidemic (urban) cycle and the sylvatic cycle described in West Africa and southern Asia (4).

Worldwide, the epidemic cycle has been the main driver of DENV circulation and transmission in recent decades. For example, all reported dengue circulation from 2010 to 2020 in Africa was linked to urban epidemics DENV 1–4 (5), and DENV-2 has been the main serotype reported in Africa for years (6). Recently, new NGS data helped to characterize six DENV-2 genotypes at the subserotype level, including VDEN-2/GVI, namely sylvatic DENV-2 (7). Its circulation and association with outbreaks in 2000 were reported from southeastern Senegal (Kedougou), but this particular genotype has circulated in Senegal since 1970 (8). In 2020, sylvatic DENV re-emerged in an outbreak with 59 recorded cases (8, 9). Previously, the most recent case of a dengue virus linked to a sylvatic strain in Africa was reported from one DHF (Dengue Hemorraghic Fever) patient from Guinea-Bissau returning to Spain in 2009 (10).

For rapid assignment of DENV serotype associated with dengue cases, many RT-qPCR systems are used globally (9, 11). In Senegal, systematic molecular serotyping of circulating DENV serotypes has been implemented since 2017 (12). This allows us to assess real-time mapping and track the changing patterns of circulating viral serotypes, which is key to public health mitigation of the dengue disease burden (5).

In November 2021, while performing molecular serotyping, we noticed serotype assignation failure using the original protocol of the Center for Disease Control and Prevention (CDC) dengue typing kit (11). For this patient sample confirmed to have dengue infection from the Saré Yoba health district, located in the Kolda region (southern Senegal), amplification curves were only observed in positive controls of the CDC dengue typing kit (13). Interestingly, the patient sample was panDENV kit positive (assay targeting DENV 3′-UTR region) (14) with a Cq (cycle threshold or crossing point) value of 26.04, indicating a detectable dengue virus RNA titer in the patient serum sample and a potential gene target failure of the CDC DENV typing kit oligos. Whole genome sequencing revealed that the virus strain unable to be assigned to a serotype by the CDC kit was a DENV-2/GVI strain (13).

To further investigate these preliminary findings and assess the link of this failure to serotype DENV-2/GVI, we retrospectively sampled six viral strains belonging to DENV-2/GVI and urban epidemic DENV 1–3 strains (*n* = 6) from the WHO collaborating center for arboviruses and hemorrhagic fever viruses at IPD (Table 1). Extracted RNA from these selected isolates (Table 1) was tested using the panDENV assay and subjected to molecular serotyping using the CDC dengue typing kit. None of the tested DENV-2/GVI RNA samples were assigned to a serotype by the CDC dengue typing kit.

**TABLE 1:** CDC dengue typing kit RT-qPCR results for used dengue strains serotyping reactions^*a*^^,^^*b*^

Sample ID	DENV serotype/ genotype	Cq PanDENV	Cq DENV-1	Cq DENV-2	Cq DENV-3	Cq DENV-4
SH381907	DENV-2/GVI	26.04	-	-	-	-
319	DENV-2/GVI	23.54	-	-	-	-
320	DENV-2/GVI	21.18	-	-	-	-
Ar578	DENV-2/GVI	21.80	-	-	-	-
Ar510	DENV-2/GVI	25.6	-	-	-	-
Ar2039	DENV-2/GVI	21.43	-	-	-	-
Ara1247	DENV-2/GVI	28.3	-	-	-	-
SH310395	DENV-2/cosmopolitan (GII)	16.21	-	13.8	-	-
SH310621	DENV-2/cosmopolitan (GII)	20.68	-	16.28	-	-
SH432820	DENV-1/GV	23.71	20.15	-	-	-
SH432822	DENV-1/GV	24.56	23.7	-	-	-
SH433217	DENV-3/GIII	32.35	-	-	27.6	-
SH433220	DENV-3/GIII	21.58	-	-	18.19	-
*PC DENV-1	DENV-1/GII	31.11	33.46	-	-	-
*PC DENV-2	DENV-2/GIV		-	31.7	-	-
*PC DENV-3	DENV-3/GV		-	-	32.62	-
*PC DENV-4	DENV-4/GI		-	-	-	31.04

^*a*^ "-" indicates no amplification**.**

^*b*^ * indicates positive controls included on the CDC dengue typing kit.

Additionally, we retrieved the genomic data corresponding to all strains tested by RT-qPCR available in GenBank. In combination with previously characterized contemporary DENV-2/GVI sequences obtained in the Kédougou region in 2020 (Table S1), we performed an *in silico* analysis of DENV-2 CDC dengue typing kit oligos to evaluate signature erosion (mismatches between viral oligos sequences against binding sites of used DENV-2 strains). Accession numbers of all used sequences during *in silico* analysis are listed in Table S1.

**Fig 1:**
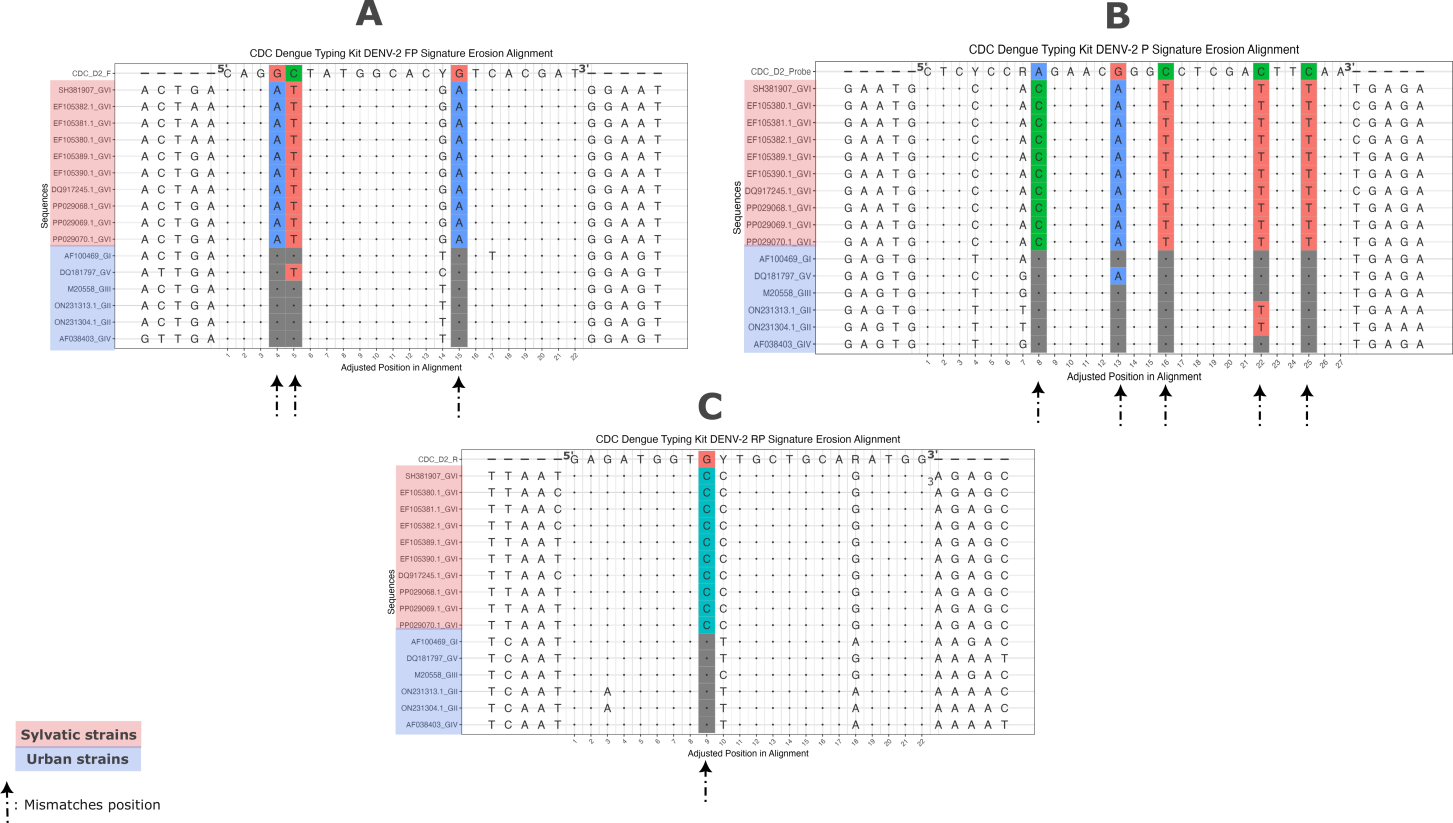
DENV-2 CDC dengue typing oligos binding sites alignment against various dengue virus genotype sequences, including DENV-2/GVI used for RT-qPCR-based viral serotyping. Panels A, B, and C represent the alignment of DENV-2 genotype sequences against forward, probe, and reverse primers of the DENV-2 of the CDC dengue typing kit, respectively. Positions with mismatches in forward, probe, and reverse primers are highlighted; conserved positions are represented by a dot. Urban (epidemic) sequence names are highlighted in blue, and sylvatic sequence names are highlighted in red. Arrows indicate the positions of mismatches of used sequences for *in silico* analysis against DENV-2 oligos included in the CDC dengue typing kit.

Obtained results unveil hotspots of mismatches of DENV-2 CDC dengue typing kit oligos against DENV-2/GVI strains in both forward primer, reverse primer, and probe binding regions (Fig. 1). While only one mismatch was observed in the forward primer binding region, and two mismatches in the reverse primer were noticed for urban dengue sequences (Fig. 1), in DENV-2/GVI sequences, three, five, and one mismatches were observed in forward, probe, and reverse primers binding sites, respectively. These mismatches are located at critical positions known to have an adverse impact on RT-qPCR reaction efficiency (15–17). The most prominent mismatch is on the 3′ end of the forward oligonucleotide, which is known to upset efficient elongation (18).

Mismatches near the 3′ end of primers are well-documented to significantly affect qPCR efficiency and often lead to higher cycle threshold (Cq) values (19, 20). The high number of mismatches (*n* = 5) observed in the probe target sequence likely affects the hybridization efficiency of the probe.

Altogether, these findings highlight that the CDC dengue typing kit oligos cannot identify re-emerging DENV-2/GVI. Previous studies highlighted the risk of sylvatic DENV-2 to spillover and the potential to cause epidemics. While the significance of sylvatic DENV-related disease in humans has been largely dismissed, we contend that such a conclusion is premature given the limited data available on sylvatic DENV infections in humans (21, 22). It has become increasingly evident that among all viruses with the potential to jump from animal reservoirs to humans, those carried by our closest relatives, such as non-human primates, are the most likely to make this transition, and sylvatic DENV is a prime example of such a virus (8, 23). Therefore, comprehensive prospective epidemiological and ecological studies in enzootic locations of Asia and West Africa are clearly a research priority (21). To allow real-time monitoring of DENV-2/GVI emergence and circulation, reliable diagnostic tools are needed. Since the target failure is limited to DENV-2/GVI strains, we propose a simple and cost-effective algorithm (Fig. S1) to assess the presence of DENV-2/GVI.

This approach might be more cost-effective than whole genome sequencing to monitor DENV-2/GVI prevalence and cryptic circulation in dengue endemic areas (Fig. S1). Real-time monitoring of this particular DENV-2 variant is important in public health in West Africa, where extensive urban dengue epidemics are increasingly being reported (12).

## SUPPLEMENTAL MATERIAL

Supplemental table and figure jcm.01741-24-s0001.docx Table S1 and Fig. S1.

ASM does not own the copyrights to Supplemental Material that may be linked to, or accessed through, an article. The authors have granted ASM a non-exclusive, world-wide license to publish the Supplemental Material files. Please contact the corresponding author directly for reuse.
