# Supplementary material for: CDC dengue typing kit fails to detect dengue virus 2 sylvatic genotype
Source: J Clin Microbiol. 2025 Jul 17;63(8):e01741-24. doi: 10.1128/jcm.01741-24 (PMC12345212; doi:10.1128/jcm.01741-24)
Supplement: Supplemental table and figure — Table S1 and Fig. S1. [file jcm.01741-24-s0001.docx]

**CDC Dengue Typing Kit Fails to Detect Dengue virus 2 Sylvatic Genotype**

^1^ Virology Unit, Institut Pasteur de Dakar

^2^ Institute of Microbiology and Virology, Brandenburg Medical School, Germany

^3^ Animal Biology Department, Université Cheikh Anta Diop de Dakar, Dakar, Senegal

**KEYWORDS:** CDC Dengue Typing Kit, DENV-2 / GVI, RT-qPCR, target failure

***** Correspondance :** [idrissa.dieng@pasteur.sn](mailto:idrissa.dieng@pasteur.sn)

^$^ This author contribute equally to this work

**Supplementary Files**

**Table S1.** Metadata of used sequences for DENV-2 CDC dengue typing kit oligos in silico analysis

| Sample ID | DENV Serotype/Genotype | Country | Collection  Year | GeneBank Accession number |
| --- | --- | --- | --- | --- |
| SH381907 | DENV-2 / GVI | Senegal | 2021 | PV614727 |
| 319 | DENV-2 / GVI | Senegal | 1999 | EF105389.1 |
| 320 | DENV-2 / GVI | Senegal | 1999 | EF105390.1 |
| Ar578 | DENV-2 / GVI | Cote d’Ivoire | 1980 | EF105380.1 |
| Ar510 | DENV-2 / GVI | Cote d’Ivoire | 1980 | EF105381.1 |
| Ar2039 | DENV-2 / GVI | Cote d’Ivoire | 1980 | EF105382.1 |
| Ara1247 | DENV-2 / GVI | Cote d’Ivoire | 1980 | DQ917245.1 |
| SH356683 | DENV-2 / GVI | Senegal | 2020 | PP029070.1 |
| SH356692 | DENV-2 / GVI | Senegal | 2020 | PP029068.1 |
| SH356702 | DENV-2 / GVI | Senegal | 2020 | PP029069.1 |
| SH310395 | DENV-2 / GII (Cosmopolitan) | Senegal | 2018 | ON231304.1 |
| SH310621 | DENV-2 / GII (Cosmopolitan) | Senegal | 2018 | ON231313.1 |
| Strain 131 | DENV-2 / GI | NA | NA | AF100469 |
| Jamaica/N.1409 | DENV-2 / GIII | NA | NA | M20558 |
| New Guinea C | DENV-2 / GIV | NA | NA | AF038403 |
| ThD2_0078_01 | DENV-2 / GV | Thailand | 2001 | DQ181797 |


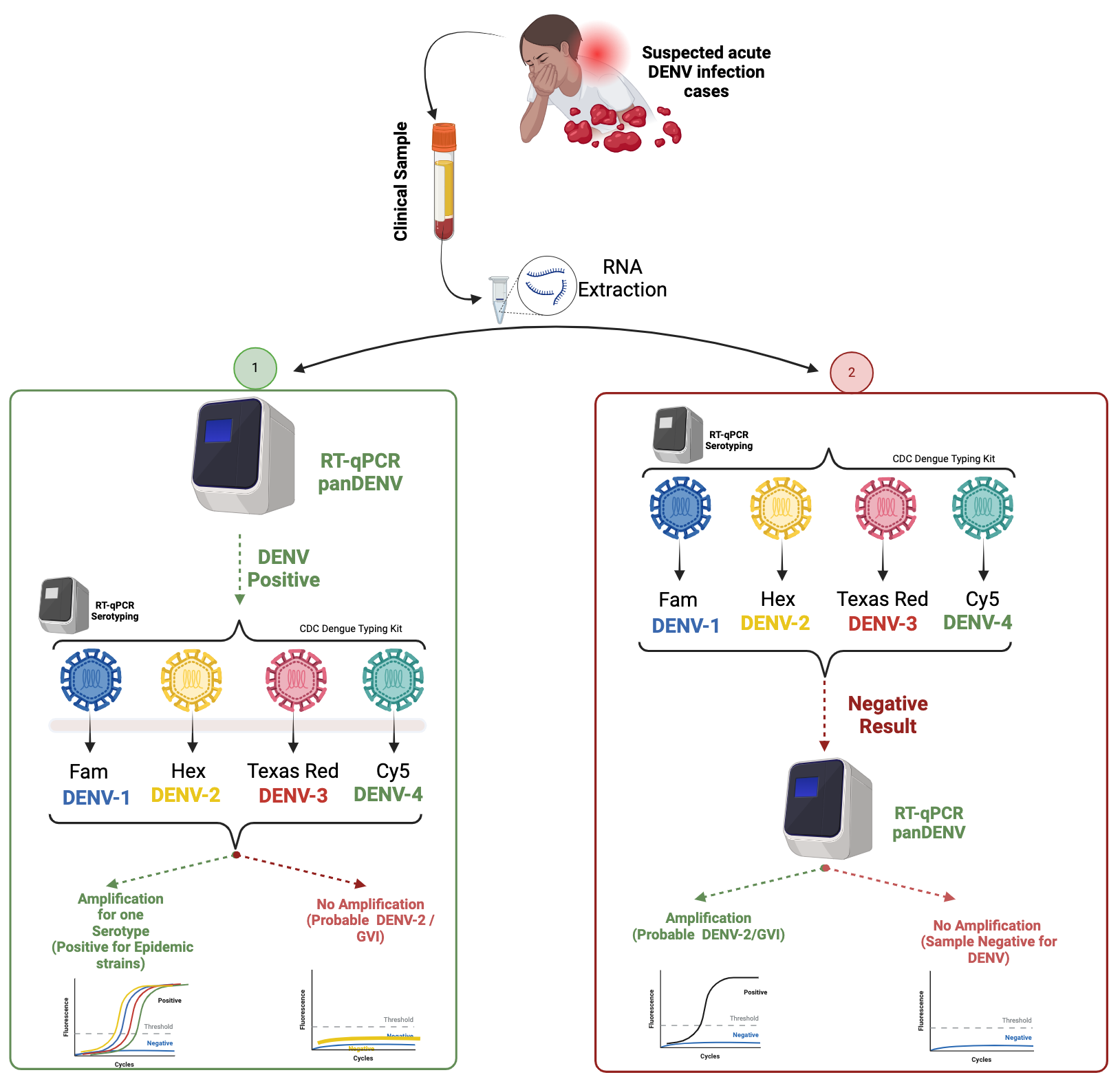


**Figure S1:** Proposed workflows leveraging DENV-2 CDC dengue typing kit gene target failure to asses DENV-2 / GVI prevalence depicted in green (marked with the number 1 inside the circle) ; In red (circle with 2) the alternate workflow. The workflow 1 is the most cost effective and recommended as primary line.
